# Replacing Forage by Crude Olive Cake in a Dairy Sheep Diet: Effects on Ruminal Fermentation and Microbial Populations in Rusitec Fermenters

**DOI:** 10.3390/ani10122235

**Published:** 2020-11-28

**Authors:** Jairo García-Rodríguez, Iván Mateos, Cristina Saro, Jesús S. González, María Dolores Carro, María José Ranilla

**Affiliations:** 1 Departamento de Producción Animal, Universidad de León, 24007 León, Spain; jgarr@unileon.es (J.G.-R.); imata@unileon.es (I.M.); csarh@unileon.es (C.S.); jsgona@unileon.es (J.S.G.); 2 Instituto de Ganadería de Montaña, CSIC-Universidad de León, Finca Marzanas s/n, 24346 Grulleros, Spain; 3 Departamento de Producción Agraria, Escuela Técnica Superior de Ingeniería Agronómica, Agroalimentaria y de Biosistemas, Universidad Politécnica de Madrid, Ciudad Universitaria, 28040 Madrid, Spain; mariadolores.carro@upm.es

**Keywords:** crude olive cake, Rusitec, dairy sheep, qPCR, ARISA, microbial protein synthesis

## Abstract

**Simple Summary:**

Large amounts of olive cake are generated after olive oil extraction, representing an environmental pollutant whose disposal is highly expensive for olive-processing companies. Using olive cake in ruminant feeding is a possible alternative, as it is a good source of fiber and contains polyphenols, although its nutritive value needs to be properly assessed. In this study, we evaluated the effects of replacing 33% of the forage (maize silage and barley straw) by olive cake (16.6% of total diet) in a mixed diet for dairy ruminants on in vitro ruminal fermentation using Rusitec fermenters. Olive cake inclusion increased diet degradability, whereas rumen fermentation, microbial growth, and microbial populations were not modified. Results indicate that maize silage and barley straw could be partially replaced by olive cake in a diet for dairy sheep without compromising rumen fermentation. The use of olive cake in ruminant diets could be especially indicated in periods of forage scarcity.

**Abstract:**

Olive oil extraction generates large amounts of a highly pollutant by-product called olive cake (OC), and its use in ruminant feeding could be an alternative. This study was designed to evaluate the effects of partially replacing forage by crude OC (COC) in a mixed dairy diet on rumen fermentation and microbial populations in Rusitec fermenters. The COC replaced 33% of the forage (66% maize silage and 33% barley straw) and was included at 16.6% of the total diet. Four fermenters were used in a cross-over design with two 13-day incubation periods. Experimental diets had a 50:50 forage-to-concentrate ratio and were formulated to contain the same protein (16.0%) and neutral detergent fiber (32.5%) levels. Compared with control fermenters, those fed the COC diet showed greater (*p ≤* 0.02) pH (6.07 vs. 6.22), diet disappearance (0.709 vs. 0.748), and butyrate proportions (18.0 vs. 19.4), but there were no differences in volatile fatty acids and ammonia production. Microbial growth, bacterial diversity, protozoal abundance, and relative abundance of fungi and archaea were unaffected by diet, although the solid phase of COC-fed fermenters showed greater (*p* = 0.01) bacterial abundance than control ones. Results indicate that COC could replace 33% of the forage in a mixed dairy diet.

## 1. Introduction

In recent years, olive oil production has experienced a great increase worldwide, with Spain being the largest producer country in the world. In 2018, Spain produced around 1.8 million tons of olive oil and more than 4.4 million tons of wet solid by-products [1], which represented around 70% of the products generated from olive processing [1]. The mechanical extraction of olive oil by the two-phase process generates a semi-solid by-product called “alperujo”, which is composed of variable proportions of olive pulp, skins, stones, and vegetative water [2]. The “alperujo” is rich in nutrients and phenolic compounds but also represents an environmental hazard [2]. In addition, as a consequence of seasonal olive production, large amounts of “alperujo” are generated in a small period of time and are stored in large ponds before being treated by partly removing the stones and drying to obtain crude olive cake (COC), a by-product that can be stored for relatively long time periods [3]. Crude olive cake can be used in ruminant feeding, but its nutritive value is low [4,5]. However, an intensive treatment to achieve greater removal of stone pieces by screening or ventilation improves its nutritional properties by reducing the neutral detergent fiber (NDF) and acid detergent fiber (ADF) content [4,5].

Crude olive cake usually has low crude protein (CP) and energy levels but high ether extract (EE), NDF, ADF, and acid detergent lignin (ADL) content [6,7]. Moreover, COC is rich in polyphenols that could improve animal health and the quality of animal products [8], although they could also negatively affect ruminal fermentation [9]. Notwithstanding, the chemical composition of COC is widely variable, especially due to the processing type and storage time [10], but also due to climate and cultivation conditions, varieties, or ripeness of olives. Even though COC has high contents of ligno-cellulose and low contents of CP and energy, it has been proposed to partly replace the concentrate [11,12], the forage [13], or even both [14,15] in diets for ruminants. It is noteworthy that some previous studies did not find negative effects of COC inclusion in the diet of ruminants not only on diet digestibility [16,17], volatile fatty acids (VFAs) production [12,13], or microbial protein synthesis (MPS) [18,19] in the rumen but also on the performance of growing lambs [13,16] and lactating sheep [11,15]. However, results are not consistent across different studies due to several factors, such as the type of diet, composition of the COC used, the ruminant species, or the level of COC inclusion, among others.

There is scarce information on the impact of COC on ruminal microbial populations and methane production. Pallara et al. [20] reported differences in the composition of bacterial populations in batch cultures when stoned COC replaced part of maize meal and barley grains in a high concentrate diet. Mannelli et al. [21] found differences in the abundance of some genera of bacteria when part of the concentrate in a mixed diet was replaced by stoned COC, although the microbial diversity in the rumen of sheep was unaffected. Our hypothesis was that partly replacing the forage in a mixed diet could modify microbial populations and methane production due to the occurrence of phenolic compounds. The present study was designed to evaluate the effects of replacing 33% of the forage by COC in a mixed diet (50:50 forage:concentrate) on in vitro rumen fermentation parameters, microbial growth, composition and diversity of bacterial populations, and the abundance of the different ruminal microbial groups in Rusitec fermenters. As far as we know, this is the first study assessing the effects of COC on ruminal microbial populations and methane production in Rusitec fermenters that allow separate collection of solid and liquid digesta.

## 2. Materials and Methods 

Sheep care and handling, as well as rumen content withdrawal, were done by trained personnel according to the instructions for experimental animal protection included in Spanish legislation (Royal Decree 53/2013), and the protocols were authorized by the competent authorities with the number ULE_014_2016.

### 2.1. Animals and Feeding

Four Merino sheep (54.2 ± 2.58 kg of BW) fitted with permanent rumen cannula were used as rumen contents donors for Rusitec fermenters. The animals received a mixed diet for 4 weeks before the start of the in vitro experiment at a fixed rate of 42 g of dry matter (DM) per kg of body weight^075^ in two meals at 09:00 and 18:00 h daily. The diet was composed of 300 g/kg DM of alfalfa hay, 240 g/kg DM of maize silage, 275 g/kg DM of maize grains, 125 g/kg DM of soybean meal, 42 g/kg DM of cottonseed meal, 8 g/kg DM of calcium soap of fatty acids, and 10 g/kg of mineral/vitamin premix. Mineral/vitamin premix was composed of 33 mg/kg of vitamin B1, 1 g/kg of vitamin E, 1.5 g/kg of niacin, 600,000 IU/kg of vitamin A, 120,000 IU/kg of vitamin D3, 2 g/kg of MnO, 300 mg/kg of IK, 4 g/kg of ZnO, 5 g/kg of S, 30 mg/kg of Na_2_SeO_3_, 1 g/kg of SO_4_Fe, 30 mg/kg of ethoxyquin, and 60 mg/kg of CoSO_4_. The chemical composition (g/kg DM) of the diet given to donor animals was organic matter (OM), 935 g; CP, 186 g; NDF, 394 g; and ADF, 179 g.

### 2.2. Experimental Diets

Two experimental mixed diets representative of those fed to dairy ruminants (forage-to-concentrate ratio 50:50) were formulated, containing 16.0% CP and 32.5% NDF. The diets differed in the composition of forage. In the control diet (CON), forage was composed of 40% alfalfa hay, 40% maize silage, and 20% barley straw, whereas forage in the COC diet contained 40% alfalfa hay, 13.4% maize silage, 13.4% barley straw, and 33.2% COC. Table 1 shows the feed ingredients and the chemical composition of both experimental diets. Forages were chopped into pieces of about 0.5 cm, and the concentrate and COC were ground at 3 mm before in vitro incubations. Maize silage and barley straw contained 973, 66, 414, 196, and 14 g/kg DM and 893, 42, 692, 365, and 40 g/kg DM of OM, CP, NDF, ADF, and ADL, respectively. The COC used in the present study had been dried and screened to eliminate olives stones pieces and it contained 893, 86.0, 404, 266, 123, 139, and 29.1 g/kg DM of OM, CP, NDF, ADF, ADL, EE, and total soluble polyphenols, respectively. The amount of nitrogen (N) insoluble in acid detergent solution (ADIN) was 48.0 of total N.

### 2.3. Rusitec Trial

The in vitro incubations were carried out using four Rusitec fermenters (effective volume of 600 mL) according to a cross-over design with two identical 13-day incubation periods. Daily management of fermenters was done as previously described by Martínez et al. [22]. The first day of the incubation, each fermenter was inoculated with solid (80 g of solid content supplied into a nylon bag) and liquid (250 mL) rumen contents from sheep collected before morning feeding, which were transferred to fermenters maintaining the temperature (30 °C) and anaerobic conditions. In addition, each fermenter received 200 mL of artificial saliva [23]. A nylon bag (100-µm pore) containing 30 g DM of diet was supplied daily into each fermenter, with the CON diet receiving two of them and the COC diet the other two. Each day, bags containing the residue were removed after 48 h of incubation and were replaced by new bags with the corresponding diet. The infusion of artificial saliva into the fermenters was maintained constantly at 665 mL/day (dilution rate 4.17% per h) to simulate the values observed in vivo in sheep [24,25]. 

After a 7-day adaptation period, fermenters were sampled for 6 days. A solution of ^15^NH_4_Cl was added to the artificial saliva (4.0 mg of ^15^N/g dietary N) from day 5 to 9 to label the bacteria present in the fermenters for quantification of MPS. The effluents collected and solid incubation residue were taken on days 8 and 9 for MPS determination following the procedure described by Carro and Miller [26]. Effluent was used to isolate liquid-associated bacteria (around 500 mL) following the procedure of Martínez et al. [22], and the remaining effluent was lyophilized and used to determine DM content and ^15^N enrichment. The solid content of nylon bags was exhaustively mixed before being used for the isolation of solid-associated bacteria and for DM and ^15^N enrichment determination [22]. Samples from both liquid and solid phases of the fermenters were also collected in sterile plastic containers, and freeze-dried before assessing microbial populations on days 8 and 9.

From day 10 to 13, the produced gas was analyzed for methane concentrations, and samples from effluent were taken for VFA and ammonia-N (NH_3_-N) analyses. In addition, the concentration of ash, NDF, and ADF in the feed residues of the incubated nylon bags were analyzed to assess the apparent disappearance of the diet after washing and drying the nylon bags according to Martínez et al. [22]. 

### 2.4. DNA Isolation and Molecular Biology Analyses

The isolation of DNA was done in triplicate from 120-mg freeze-dried samples of liquid and solid, using a procedure optimized for DNA extraction from digesta and fecal samples [27] with an added step with cetyltrimethylammonium bromide (CTAB) for removal of PCR inhibitors [28]. DNA quantification and the evaluation of its purity from the absorbance ratios (A260:A280 and A260:A230) were carried out in a Nanodrop-ND-1000 (NanoDrop Technologies, Wilmington, DE, USA), with the values of A260:A280 ranging from 1.84 to 1.98 and those of A260:A230 from 1.65 to 2.24, so it can be considered a good DNA quality.

Quantitative PCR (qPCR) was carried out in duplicate to quantify bacterial and protozoal DNA concentrations. Additionally, the relative abundance of archaea and fungi was calculated in relation to total bacteria. This technique was performed in an ABI PRISM 7000 Sequence Detection System (Applied Biosystems, Warrington, UK) as described by Saro et al. [28]. For general bacteria and fungi, the primers used were those defined by Denman and McSweeney [29]. For protozoa, the primers used were described by Sylvester el al. [30] and those used for methanogenic archaea by Denman et al. [31]. Rumen bacterial and protozoal DNA used as a standard was extracted from microbial pellets previously isolated from the rumen of sheep [28].

As described by Saro et al. [32], the internal transcribe spacer of DNA was amplified for ARISA analysis using 16S-1392F and 23S-125R universal primers [33] and a 2720 Thermal Cycler (Applied Biosystems, Foster City, CA, USA). The amplified PCR product was resolved in an ABI Prism 3130 Genetic Analyzer (Applied Biosystems) for the automated detection of ARISA fragments. The resulting data were analyzed using the software GeneMaker v 1.80 (SoftGenetics, State College, PA, USA), where peaks were compared with an internal size standard for their determination. The comparison of the profile of the electropherograms was carried out by a dissimilarity matrix using the presence or the absence of the different peaks. A principal coordinate analysis (PCoA) was done using the R package vegan [34] to evaluate the differences among samples. This PCoA was based on the Bray–Curtis dissimilarities.

### 2.5. Analytical Procedures

The analyses of DM (ID 934.01), ash (ID 942.05), N (ID 984.13), and ether extract (EE; ID 945.16) concentrations were conducted according to the Association of Official Analytical Chemists (AOAC) [35]. Analysis of NDF, ADF, and LAD were performed as described by Van Soest et al. [36], using an ANKOM220 Fiber Analyzer unit (ANKOM Technology Corporation, Fairport, NY, USA). Sodium sulphite and heat-stable amylase were used in the analysis of NDF. Results of NDF and ADF were expressed exclusive of residual ash. Procedures for the determination of total soluble polyphenols and ADIN content have been described by Marcos et al. [7].

Analyses of VFA and NH_3_-N were performed following the procedures of Martínez et al. [22]. The gas produced from the fermenters was analyzed by gas chromatography for methane determination according to Martínez et al. [37]. The synthesis of microbial protein was quantified following the procedure described by Carro and Miller [26], using ^15^N as microbial marker.

### 2.6. Calculations and Statistical Analyses

The amount of OM apparently fermented was calculated from VFA production as described by Demeyer [38], and it was used to estimate the efficiency of MPS. Shannon’s diversity index [39] was used to assess the diversity of the bacterial communities in the fermenters. The relative abundance of archaea and fungi was estimated relative to the absolute bacterial abundance as follows: 2^―(CT target―CT total bacteria)^. C_T_ is the value of the threshold cycle previously corrected for differences in the efficiency of amplification among the target (archaea or fungi) and the reference (bacteria) The values of the correction factor were 1.005 for fungi and 1.059 for archaea. 

A mixed model including repeated measures was used to analyze the data on fermentation parameters and diet disappearance. Diet, time, diet x time interactions, and incubation run were included as fixed effects in the statistical model, while the fermenter was considered a random effect. Statistical analysis of the data on molecular biology and microbial growth was done as a mixed model, which included diet and incubation run as fixed effects, and fermenter as a random effect. The MIXED procedure of SAS (SAS Institute Inc., Cary, NC, USA) was used for statistical analyses. Effects were considered significant at *p* < 0.05, and *p* < 0.10 was considered a trend.

## 3. Results and Discussion

### 3.1. Effect of Crude Olive Cake (COC) Inclusion on Diet Disappearance and Rumen Fermentation Parameters

The chemical composition of the COC used in this study was in the range of values previously reported [5,6,7,10], and the previous screening of this sample agrees well with its low content in NDF and ADF compared with other COC samples [7,10]. In accordance with other studies [6,7,10], a high proportion (48.0%) of N in COC was linked to ADF, indicating a low N availability. 

The values of the main fermentation parameters and diet disappearance for both experimental diets are shown in Table 2. Higher pH values (*p* = 0.02) were found in COC fermenters compared to CON ones. Previous in vivo studies observed that replacing up to 20% of concentrate in steers [12] or up to 67% of forage in lambs [13] fed high-concentrate diets by COC did not affect rumen pH values. Diet did not affect (*p* = 0.24) daily NH_3_-N production, and for both diets, concentrations were greater than 50 mg NH_3_-N per liter established as the minimum threshold for optimal microbial protein production in vitro [40]. In agreement with our results, Estaún et al. [12] found no effects on NH_3_-N concentration when exhausted olive cake replaced 20% of concentrate in steers fed a high-concentrate diet. However, replacing 33% and 67% of forage by COC in a high-concentrate diet for growing lambs resulted in greater and lower NH_3_-N concentrations compared to a control diet, respectively [13]. Ammonia-N is the end-product of microbial N metabolism in the rumen, and its concentration depends on the balance between the release of NH_3_-N from the degradation of CP and its caption by ruminal microorganisms for their growth. The discrepancies observed among studies in NH_3_-N production when olive cake is included in the diet suggest that the amount included in the diet, the ingredients replaced, and the type of olive cake markedly influence the results.

Total VFA daily production was not affected (*p* = 0.19) by the presence of COC in the diet, which is positive since VFA can contribute up to 70% of the energy requirements of ruminants [41]. In agreement with our results, previous in vivo studies found that replacing up to 20% of concentrate in steers [12] or up to 67% of forage in lambs fed high-concentrate diets [13] did not have any effect on VFA concentrations in the rumen. In contrast, the VFA profile was modified by COC inclusion, resulting in reduced acetate (*p* = 0.01) and caproate (*p* < 0.001) proportions, increased proportions of butyrate (*p* = 0.001) and isovalerate (*p* < 0.001), and no changes (*p* = 0.22) in the acetate/propionate ratio. Information on the effects of COC inclusion in the diet on the VFA profile is limited, but previous studies reported high proportions (>61.0%) of acetate in the in vitro fermentation of different types of olive cake [7,10], which is consistent with its high NDF content. Pallara et al. [20] observed that replacing part of maize meal and barley in a high-concentrate diet by 90 g/kg DM of stoned COC increased propionate, butyrate, and isovalerate production, and reduced the acetate/propionate ratio in batch cultures. 

Daily methane production tended (*p* = 0.08) to be lower in COC fermenters compared to CON ones, but the methane/VFA ratio was unaffected (*p* = 0.67) by the diet. There is no information about the effects of including COC in ruminant diets on methane production, but Shakeri et al. [42] reported that in vitro fermentation of olive pulp and skins resulted in 12% and 22% lower gas and methane yield, respectively, compared to oaten chaff. The high EE content of the COC used in our study and the presence of polyphenols might have contributed to the reduced methane production observed in COC-fed fermenters, as both compounds can decrease methane production [43]. The potential effects of COC on methane production probably depend on the type and composition of olive cake and its level of inclusion in the diet, but more studies are needed to clarify this issue. 

The COC diet showed greater (*p* < 0.001) values of DM, OM, NDF, and ADF disappearance compared to the CON diet (Table 2). The higher disappearance observed for the COC diet is in contrast to previous studies, which reported no effects [16,17] or a reduction [14] of in vivo DM, OM, NDF, and ADF apparent digestibility when COC was included in the diet of growing lambs up to 150 g/kg DM. Similarly, Farghaly et al. [13] observed that the replacement of 67% of clover hay by COC reduced DM, OM, and ADF apparent digestibility in growing lambs, although that of NDF was unaffected. Yáñez-Rúiz and Molina-Alcaide [44] reported a reduction in DM, OM, NDF, and ADF digestibility in both wethers and goats fed alfalfa hay when exhausted olive cake (12.8%) was included in the diet. The lack of differences between diets in VFA production in our study contrasts with the increased disappearance values observed for the COC diet, and it might be partly explained by losses of small COC particles through the pores of the nylon bags, as the COC used in our study had been screened and had a small particle size. In addition, the COC sample has a lower NDF and ADF content than those used in the previously cited studies, and therefore greater digestibility could be expected. As already discussed, the contrasting results observed among studies assessing the effects of COC on ruminal fermentation might be related to different factors, such as the type and composition of the olive cake used, the fermentation system (in vitro or in vivo), the diet composition, the level of inclusion, and the dietary ingredients that were replaced, among others. In brief, the results of this study suggest no negative effects of the used COC on ruminal fermentation when 16.6% of the forage was replaced in a mixed diet. 

### 3.2. Effects of Crude Olive Cake (COC) Inclusion on Microbial Protein Synthesis (MPS), Bacterial Diversity, and Microbial Populations

The results of MPS and its efficiency are shown in Table 3. There were no effects of diet on total MPS and its efficiency (*p* = 0.15 and 0.79, respectively), although MPS in the solid phase was lower (*p* = 0.05) and that in the liquid phase greater (*p* = 0.03) in COC fermenters compared with those receiving the CON diet. Microbial protein is essential for ruminants since it can supply from 60% to 85% of the amino acids reaching the small intestine [45]. The lack of effects of COC inclusion on total MPS and its efficiency indicates that microbial N supply for the animal would be not negatively affected. To our best knowledge, this is the first study in which MPS and its efficiency were assessed using ^15^N as an external marker when COC is included in a diet for ruminants. In agreement with our results, Molina-Alcaide et al. [19] observed that replacing 40% of forage by a concentrate containing 66% barley grains and 34% exhausted olive cake in an alfalfa hay-based diet (140 g of COC/kg diet DM) resulted in no differences in MPS and its efficiency in continuous fermenters using DAPA as a microbial marker. Similarly, Abbedou et al. [18] did not observe differences in the efficiency of MPS, estimated from daily urinary allantoin and purine, when 70% of concentrate was replaced by COC in a 50:50 forage concentrate diet for sheep. In contrast, Yáñez-Rúiz et al. [46] analyzed the effects of including 18.8% COC in the diet of wethers and goats fed only alfalfa hay, and observed a significant reduction in the MPS estimated from urinary excretion of allantoin in wethers, but no changes were detected in goats. Allantoin is the main purine derivative in the urine of small ruminants and its urinary excretion is used to estimate MPS in ruminants [46].

The effects of the experimental diets on bacterial diversity and microbial populations in Rusitec fermenters are shown in Table 4. In the solid phase of the digesta, the quantity of DNA of bacteria was greater (*p* = 0.01) and that of protozoa tended to be greater (*p* = 0.06) in COC-fed fermenters compared to CON ones, which agrees well with the higher pH found in fermenters that received the COC diet, as a decrease in pH compromises the growth of some bacteria and protozoa [47]. The higher bacterial and protozoal DNA concentrations found in the solid digesta of COC fermenters disagrees with the reduction of MPS caused by the COC diet in this digesta phase (Table 3). The lower MPS could be due to a lower amount of incubation residue in the nylon bags of COC fermenters, as indicated by the greater disappearance of diet DM. Thus, the PCR results indicate that feed particles of the COC diet had a greater density of bacteria and protozoa, but the total amount of microbial protein synthetized was lower than in CON fermenters due to the lower amount of incubation residue. No differences (*p* ≥ 0.17) between diets were detected either in the relative abundance of fungi and archaea in the solid phase or in any of the examined microbial groups in the liquid phase of fermenters. Bacterial diversity was also unaffected by diet in both the solid (*p* ≥ 0.56) and liquid (*p* ≥ 0.81) phases.

**Figure 1:**
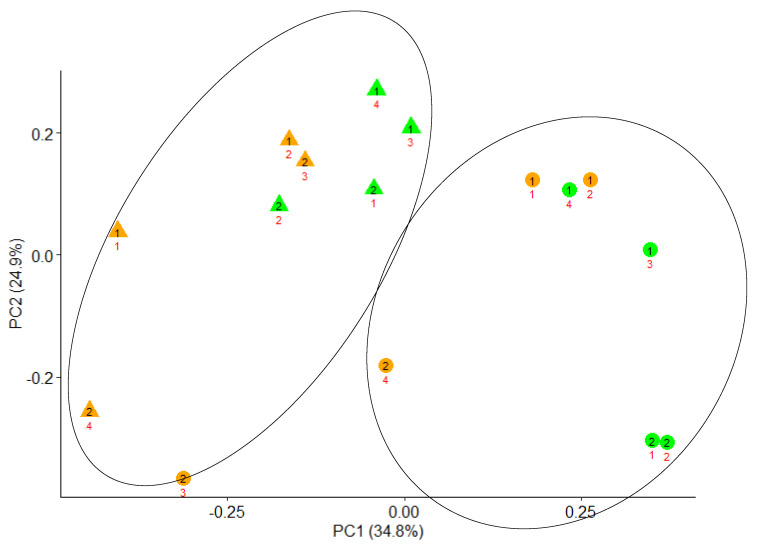
Principal coordinate analysis (PCoA) plot (Bray–Curtis dissimilarities) of the automated ribosomal intergenic spacer analysis (ARISA) profiles from digesta of Rusitec fermenters fed either a mixed diet (orange) or a diet containing crude olive cake (green) replacing 33% of the forage. Circles and triangles correspond to liquid and solid digesta, respectively. Numbers in black (1 and 2) relate to incubation run, and those in red (1 to 4) to a Rusitec fermenter.

The PCoA plot based on the Bray–Curtis distances is represented in Figure 1. As can be observed in the plot, 34.8% and 24.9% of the variance was explained by the principal coordinates 1 and 2, respectively. The PCoA plot showed no clear separation of samples according to the diet, but they were grouped according to digesta phase (solid or liquid). Thus, the results of ARISA of this study show that diet did not affect either the bacterial diversity or the structure of bacterial communities, determined by the similarity or difference among samples due to the different species (or OTUs) that composed them, and defined as the composition of a bacterial community and the abundance of its members. Olive cake contains phenolic compounds—including tannins, phenolic alcohols, phenolic acids, and flavonoids [6,48]—which might affect the microbial populations in the rumen [49], and could favorably modify rumen fermentation through dose-dependent and specific effects on microorganisms [50]. Previous studies reported effects of olive cake polyphenols on the structure of bacterial communities, but no negative effects were observed on bacterial diversity, defined as the number of different bacterial species present in the rumen. Cappucci et al. [51] supplemented a diet for dairy sheep with 1.2 g/kg DM of an olive crude phenolic concentrate and they did not find any effect on bacterial diversity in the rumen fluid after 5 weeks of treatment, but the structure of rumen bacterial communities appeared to be different as indicated by the analysis of total bacterial DGGD (denaturing gradient gel electrophoresis) fingerprints. Mannelli et al. [21] included 135 g/kg DM of stoned COC in a concentrate for sheep and they did not observe any effect on microbial diversity in rumen fluid, although metataxonomic analysis showed changes on some bacterial genera that suggested a specific antimicrobial activity of COC. In the same way, Pallara et al. [20] observed that replacing part of maize meal and barley in a high-concentrate diet by 50 and 90 g/kg DM of stoned COC resulted in a different structure and composition of bacteria populations in batch cultures. Thus, results from previous studies [20,21,51] support the lack of effects on bacterial diversity when COC was included in the diet, although the effect on the structure of bacterial communities appears to be variable. In summary, in our study, there were no detrimental effects of partly replacing the forage in a mixed diet by COC on microbial populations, bacterial diversity, and the structure of bacterial communities in Rusitec fermenters. 

## 4. Conclusions

Results of the present study indicate that COC can replace 33% of the total forage (66% of maize silage and 33% of barley straw) in a mixed diet without producing marked changes in rumen fermentation and without affecting the synthesis of microbial protein. The incorporation of COC increased diet disappearance, tended to decrease methane production, and produced only subtle changes in microbial populations. The partial replacement of forage by COC in ruminant diets might be especially useful in periods of limited forage availability and would allow a reduction of forage crops in favor of other crops for human or animal consumption.

## Figures and Tables

**Table 1:** Ingredients and chemical composition of the experimental diets used in the Rusitec fermenters.

Item	Diet	
	CON	COC
**Ingredients (g/kg DM)**		
Alfalfa hay	200	200
Maize silage	200	67
Barley straw	100	67
Olive cake	-	166
Soybean meal	147	147
Extruded maize	134	134
Barley	200	200
Mineral/vitamin premix ^1^	10	10
Calcium soap of fatty acids	9	9
**Chemical composition (g/kg DM)**		
Organic matter	943	929
Crude protein	161	160
Neutral detergent fiber ^2^	324	322
Acid detergent fiber ^2^	163	155
Ether extract	28.2	45.0

^1^ Declared composition (g/kg mineral/vitamin premix): vitamin A, 600,000 IU; Vitamin E, 1 g; Vitamin B1, 33 mg; Vitamin D3, 120,000 IU; Niacine, 1.5 g; S, 5 g; ZnO, 4 g; IK, 300 mg; SO4Fe, 1 g; MnO, 2 g; Na2SeO3, 30 mg; CoSO4, 60 mg; Ethoxyquin, 30 mg. ^2^ Expressed exclusive of residual ash.

**Table 2:** Effects of partially replacing maize silage and barley straw in a mixed diet (CON) by crude olive cake (COC) on ruminal fermentation parameters and diet disappearance in Rusitec fermenters (*n* = 4).

Item	Diet		SEM	*p*-Value
	CON	COC		
**Fermentation parameters**				
pH	6.07	6.22	0.088	0.02
Ammonia-N (mg/d)	123	142	9.7	0.24
Total volatile fatty acids (mmol/d)	94.1	91.5	2.71	0.19
Molar proportions (mol/100 mol)				
Acetate	47.8	46.9	0.50	0.01
Propionate	21.3	21.3	0.91	0.99
Butyrate	18.0	19.4	0.53	0.001
Isobutyrate	0.63	0.52	0.204	0.46
Isovalerate	1.93	2.48	0.124	<0.001
Valerate	5.79	5.78	0.167	0.89
Caproate	4.5	3.73	0.185	<0.001
Acetate/propionate (mol/mol)	2.35	2.22	0.103	0.22
Methane (mmol/d)	23.3	22.0	0.99	0.08
Methane/Total VFA (mol/mol)	0.247	0.249	0.0060	0.67
**Diet disappearance (g/g)**				
Dry matter	0.709	0.748	0.0085	<0.001
Organic matter	0.699	0.737	0.0087	<0.001
Neutral Detergent Fiber	0.259	0.403	0.0133	<0.001
Acid Detergent Fiber	0.182	0.282	0.0160	<0.001

**Table 3:** Effects of partially replacing maize silage and barley straw in a mixed diet (CON) by crude olive cake (COC) on microbial protein synthesis (in solid and liquid digesta) and on its efficiency in Rusitec fermenters (*n* = 4).

Item	Diet		SEM	*p*-Value
	CON	COC		
Microbial protein synthesis (mg N/day)				
Solid phase	199	171	7.2	0.05
Liquid phase	107	119	2.6	0.03
Total	306	290	6.3	0.15
Efficiency of microbial growth ^1^	32.9	32.6	0.81	0.79

^1^ Expressed as mg microbial N per g OM apparently fermented; OM apparently fermented was estimated from VFA production according to Demeyer [38].

**Table 4:** Effects of partially replacing maize silage and barley straw in a mixed diet (CON) by crude olive cake (COC) on microbial abundance and bacterial diversity in solid and liquid phases of Rusitec fermenters (*n* = 4).

Digesta Phase	Item ^1^	Diet		SEM	*p*-Value
		CON	COC		
**Solid**	Total bacteria ^2^	101	193	12.2	0.01
	Total protozoa ^2^	0.0015	0.0067	0.00106	0.06
	Fungi ^3^	46.7	36.6	19.33	0.80
	Archaea ^2^	0.03	0.05	0.006	0.36
	Number of peaks	21.0	22.5	0.85	0.56
	Shannon index	3.04	3.09	0.029	0.66
**Liquid**	Total bacteria ^2^	1.75	2.01	0.246	0.72
	Total protozoa ^2^	0.0002	0.0002	0.00002	0.99
	Fungi ^3^	0.006	0.015	0.0022	0.25
	Archaea ^2^	0.02	0.39	0.156	0.17
	Number of peaks	29.2	30.2	1.06	0.81
	Shannon index	3.37	3.39	0.093	0.85

^1^ Abundance of bacteria and protozoa DNA and the relative abundance of fungi and archaea were determined by qPCR, and bacterial diversity was analyzed by automated ribosomal intergenic spacer analysis (ARISA); ^2^ Expressed as µg DNA/g dry matter in the solid phase and as µg DNA/mL in the liquid phase; ^3^ Expressed as relative abundance to the absolute quantification of total bacteria as 2^―(*CT* target*―CT* total bacteria)^.
